# Comparison of DNA Extraction Methods and Real-Time PCR Assays for the Detection of *Blastocystis* sp. in Stool Specimens

**DOI:** 10.3390/microorganisms8111768

**Published:** 2020-11-11

**Authors:** Céline Nourrisson, Julie Brunet, Pierre Flori, Maxime Moniot, Virginie Bonnin, Frédéric Delbac, Philippe Poirier

**Affiliations:** 1 Service de Parasitologie-Mycologie, CHU Clermont-Ferrand, 3iHP, 63000 Clermont-Ferrand, France; drbrunet.julie@gmail.com (J.B.); mmoniot@chu-clermontferrand.fr (M.M.); ppoirier@chu-clermontferrand.fr (P.P.); 2 Laboratoire Microorganismes, Génome et Environnement, CNRS, Université Clermont Auvergne, 63178 Aubière, France; frederic.delbac@uca.fr; 3 Service des Agents Infectieux et Hygiène, Unité de Parasitologie-Mycologie, CHU Saint-Etienne, 42270 Saint-Priest-en-Jarez, France; pierre.flori@chu-st-etienne.fr; 4 Microbes, Intestin, Inflammation et Susceptibilité de l’Hôte, INSERM, USC INRA, Université Clermont Auvergne, 3iHP, 63000 Clermont-Ferrand, France; virginie.bonnin@uca.fr

**Keywords:** *Blastocystis*, qPCR diagnosis, fecal microbiota transplantation

## Abstract

Diagnosis of *Blastocystis* in stool may be challenging, as microscopic examination and culture-based methods have demonstrated low sensitivity. Molecular detection assays are now available for this enteric parasite, based on “in-house” or commercial-developed techniques. The aim of this study was to assess and compare the performance of (i) two DNA extraction methods (manual versus automated), and (ii) four qPCR assays (three “in-house” and one commercialized), for detection of *Blastocystis* sp. in human stools. One hundred and forty stools were included, among which 76 were confirmed to be positive for *Blastocystis*. The manual DNA extraction method allowed for the identification of significantly more positive specimens than the automated method (*p* < 0.05). In particular, specimens with a low parasite load were negative when DNA was extracted with the automated process. The four qPCR assays also had variable performances, with the commercialized assay being the most sensitive (84%) but the least specific (82%). Overall, for all qPCR assays, the specificity decreased when the sensitivity increased. *Blastocystis’* subtype, notably the subtype 4, influenced these performances. Our results indicate that the positivity rate for the detection of *Blastocystis* in stools could be variable according to the DNA extraction method and the qPCR assay used. These pitfalls need to be considered for the selection of method and interpretation of results, particularly considering the search of this intestinal parasite in a donor before fecal microbiota transplantation.

## 1. Introduction

*Blastocystis* is the most common human intestinal parasite, infecting probably more than 1 billion people in the world. Twenty-two subtypes (STs) of the parasite have been described using a 600 bp barcode sequence of the 18S rRNA encoding gene [1]. Among them, ST1 to ST9 and ST12 were identified in human stools, with ST1 to ST4 being the most frequent [2,3]. Two parasitic forms, namely vacuolar and cyst forms, can be detected in the stools of infected subjects. The pathogenic potential of *Blastocystis* is unclear, as most patients infected with this parasite are asymptomatic [4]. However, several studies have shown a higher prevalence of *Blastocystis* in patients suffering from irritable bowel syndrome, and recent data have suggested that it could be associated with fecal microbiota modifications [5,6,7,8,9]. Moreover, we recently demonstrated in an animal model that *Blastocystis* establishment was associated with visceral hypersensitivity and microbiota changes [10]. So, the impact of *Blastocystis* on human health remains debated, explaining the inclusion of *Blastocystis* among the parasites to be screened for stool qualification before fecal microbiota transplantation (FMT) [11]. Indeed, cases of transmission of ST1 and ST3 were recently reported in recipients after FMT from positive donors [12].

Several studies have shown that molecular diagnosis using PCR is the most sensitive tool when compared to direct light microscopy (DLM) or culture-based methods [13]. Four in-house qPCR techniques have been previously published, of which three target the 18S rRNA encoding gene [13,14,15,16]. Some multiplex PCR assays that include *Blastocystis* in their panel are also commercialized. These methods often recommend swab sampling of stool and conservation in a specific medium before DNA extraction. Considering that medical laboratories need an accurate diagnosis for *Blastocystis*, particularly for donor screening prior to FMT, the aim of our study was to compare (i) two methods for DNA extraction from stools and (ii) three in-house qPCR assays and one commercial kit for detection of *Blastocystis* in human stools.

## 2. Materials and Methods

### 2.1. Specimens

Stool samples were collected from the parasitology medical laboratory of the teaching hospital of Clermont-Ferrand (France) and submitted for routine parasitic analysis (including direct light microscopy (DLM)) between July 2014 and December 2015. All patients were informed of the potential use of their stool for research applications; in case of refusal, they had to provide an opposition form, as validated by the ethics committee of our institution (university and hospital center of Clermont-Ferrand, France). The goal of the recruitment was to obtain a maximum number of positive stools for *Blastocystis* sp. So, patients with recent travel or infected with other enteric parasites or whose stool was identified as being positive for *Blastocystis* with DLM were systematically enrolled [17].

### 2.2. DNA Extraction

The day of sampling, DNA was extracted from 200 mg of stools after a bead beating step (30 m/s during 3 min) with the non-automated QIAamp DNA Stool Minikit (Qiagen, Venlo, The Netherlands), according to the manufacturer’s instructions, with a final elution volume of 200 µL. In parallel, flocked nylon swabs (ESwab, Copan Diagnostics Inc, Murrieta, CA, USA) were used to sample stools according to the manufacturer’s recommendations, which stated that the swab should be dipped in the stool, then the excess stool on the swab should be removed before discharge of the swab into the transport medium (1200 µL) by vortexing. Transport media were stored at −80 °C until DNA extraction. Transport media were then thawed, vortexed, and the totality was extracted on the QIAsymphony^®^ DNA extractor (Qiagen, Venlo, The Netherlands), with a final elution volume of 85 µL. All DNA extracts were stored at −80 °C until qPCR analysis.

DNA from rare *Blastocystis* subtypes (ST5, ST6, ST8, and ST9) were used for PCR comparison. DNA from ST5 and ST9 were kindly provided by Dr. Christen Rune Stensvold (Statens Serum Institut, Copenhagen, Denmark).

### 2.3. Comparison of the Four qPCR Assays

Three published qPCR assays targeting the 18S rRNA encoding gene were tested in our study on the Rotor-Gene^®^ Q (Corbett Life Science Pty. Ltd., Sydney, Australia) according to previously described protocols [13,15,16]. All sample extracts were checked for the presence of PCR inhibitors by mixing an equal volume of the DNA extracts and the positive control (personal positive DNA for *Blastocystis*). Mixed samples with a Ct value greater than the Ct of positive control alone (2-fold diluted) were tested in qPCR again after a 10-fold dilution. We also tested the Allplex^TM^ Gastrointestinal Panel-Parasite Assay (Seegene, Seoul, Korea) on a CFX96^TM^ real-time PCR detection system (BioRad, Marnes-la-Coquette, France) according to the manufacturer’s instructions. This multiplex assay is certified CE-IVD (this marking indicates that the in vitro diagnostic device considered complies with the European in vitro diagnosic devices directive) and can detect six eukaryotic parasites simultaneously: Giardia intestinalis, Entamoeba histolytica, Cryptosporidium spp., *Dientamoeba fragilis, Cyclospora cayetanensis*, and *Blastocystis* spp. An internal control is incorporated to check for possible PCR inhibitors.

### 2.4. *Blastocystis* Subtype Sequencing

Sequencing was performed for each sample when it was positive in at least one of the four qPCR assays. Primers for sequencing were preferentially those used in the qPCR method developed by Poirier et al. [13]. In cases where this qPCR assay was negative, primers used for sequencing were those of positive qPCR assays. The PCR product was purified with a NucleoSpin^®^ extract kit (Macherey-Nagel, Düren, Germany) and then sequenced by Eurofins Genomics (Köln, Germany). Sequences were analyzed using the Basic Local Alignment Search Tool (BLAST) (https://blast.ncbi.nlm.nih.gov). On the basis of qPCR products obtained with the Poirier et al. [13] method, subtypes were assigned with a query coverage >98% with exact match or identity >98%. The qPCR products obtained with the other assays did not allow for discrimination between subtypes, so in these cases, the final result was recorded as “*Blastocystis* sp.”.

Each sample for which a sequence corresponding to “*Blastocystis*” was obtained was considered a ‘true positive’ sample. Each sample for which no sequence corresponding to “*Blastocystis*” was obtained despite a positive qPCR was considered a ‘false positive’ sample for this qPCR assay.

### 2.5. Statistical Analysis

Statistical analysis was performed with XLSTAT software (version number 2018.5, Addinsoft Inc, New York, NY, USA). The sensitivity and specificity of the four qPCR assays were calculated and compared to sequencing results as the gold standard.

## 3. Results

### 3.1. Specimens

One hundred and forty stool specimens were included in the study. All stools were first observed by direct light microscopy (DLM) and analyzed with the four different qPCR assays. All stools positive with DLM (*n* = 26) were also positive in qPCR. All positive stools with at least one of the four qPCR methods were then confirmed by sequencing. Overall, 76 stools were confirmed to be ‘true positive’ among the 140 specimens and were used to assess the performance of the various methods (Table S1).

### 3.2. Comparison of DNA Extraction Procedures

**Figure 1:**
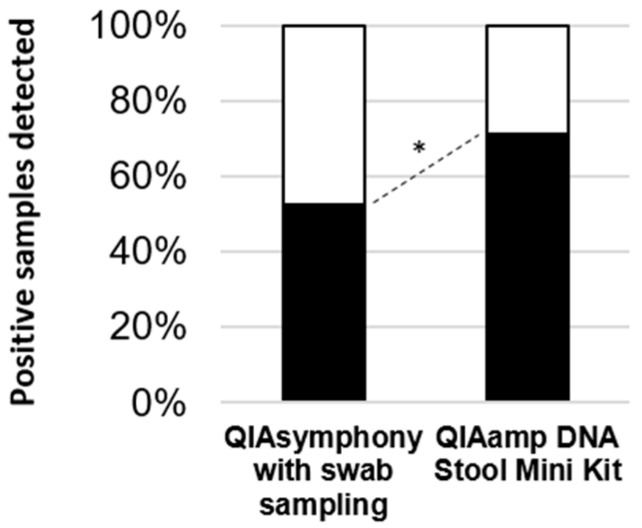
Comparison of DNA extraction methods using the qPCR assay developed by Poirier et al. Positive samples detected using the qPCR published by Poirier et al. [12] are represented in black and positive samples not detected are in white. *: *p* < 0.05.

DNA from the 140 stool specimens were extracted with both manual and automated methods. The comparison between both procedures was performed with two in-house qPCR methods: one was developed by Poirier et al. in 2011 [13] and the second by Stensvold et al. in 2012 [14]. When considering both qPCR assays associated with the manual extraction, 54/76 and 60/76 samples were positive, respectively. For both qPCR assays, the use of automated DNA extraction was associated with a significant loss of sensitivity, with 40/76 and 26/76 positive samples, respectively (Yates correction of chi-squared: *p* < 0.05; the results of Poirier’s qPCR are shown in Figure 1). For the false negative specimens with automated extraction (i.e., positive when manually extracted), the mean Ct value of these specimens when manually extracted was 34.37 ± 5.05 versus 19.38 ± 5.93 (Student’s *t*-test: *p* < 0.001) for other positive specimens when using Poirier et al.’s qPCR, and 26.54 ± 9.67 versus 19.48 ± 5.58 (Student’s *t*-test: *p* = 0.002) when using Stensvold et al.’s qPCR. The ST had no influence on the results, regardless of the extraction method used (data not shown).

Considering these results, a comparison of the four qPCR assays was only performed on DNA extracts obtained with the manual technique.

### 3.3. Comparison of the Four qPCR Assays

The sensitivity and specificity of each qPCR method and DLM are reported in Table 1.

The marketed qPCR was the most sensitive but the least specific. On the contrary, DLM was highly specific (100%) but lacked sensitivity (34%). Overall, when sensitivity increased, the specificity decreased.

**Figure 2:**
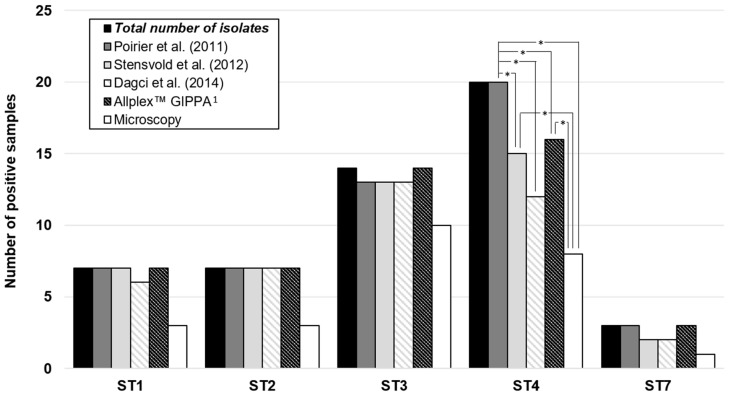
Detection of *Blastocystis* according to subtypes and diagnosis methods. The total number of positive isolates for each subtype, all methods included, is represented in dark. Each different method is also represented. *: *p* < 0.05. ^1^ Gastrointestinal Panel-Parasite Assay.

Concerning subtypes, the detection of ST4 was significantly lower with DLM and also with all of the qPCR techniques with the exception of the qPCR developed by Poirier et al. (*p* < 0.05; Figure 2). The detection of the other STs was not different according to the qPCR assay. All of the qPCR assays also detected rare STs (ST5, ST6, ST8, and ST9, data not shown) that can infect humans.

## 4. Discussion

The objective of this study was to compare various DNA extraction procedures from stools and qPCR assays targeting *Blastocystis* in order to determine their applicability for medical use and especially FMT safety screening. Our results confirmed the poor sensitivity of microscopic examination, even for high parasitic loads, as shown in previous studies [13]. Importantly, the present study highlighted the important variability between DNA extraction methods and qPCR molecular assays for diagnosis of this enteric pathogen.

For the DNA extraction step, positive samples with the lowest parasite load were not detected when the extraction was performed with the automated method. This could be due to the amount of the extracted stool, as swab sampling induces stool dilution compared to the manual method using 200 mg of stool. The same automated extraction technique has previously been evaluated, including for different parasites, with acceptable performances [18,19]. We used the same sampling device as described by Laude et al., but their initial sampling of 200 mg was superior to ours on the swab, which could explain our lower performances [18]. In addition, the bead beating step was not recommended in the protocol of automated extraction. *Blastocystis*’s cyst form, like other protozoans, is surrounded by a thick wall that can resist classic lysis. However, even for stools containing vacuolar forms of *Blastocystis*, some qPCR assays were negative when using the automated extraction. Finally, this discrepancy could also be explained by the tandem between extraction method and qPCR assay. Indeed, as previously described for other pathogens, it is essential to check the correct match between the DNA extraction and the PCR method [20]. This can be observed in our study, as we found for manual extraction a sensitivity of 79% for the qPCR published by Stensvold et al. and 71% for the method developed by Poirier et al. However, the Poirier et al. method detected significantly more positive samples (53%) with automated extracts compared to the qPCR published by Stensvold et al. (34%). So, the combination of automated extraction with the qPCR published by Poirier et al. was more efficient than the combination with the Stensvold et al. qPCR.

We then compared three in-house qPCR assays and one commercial assay. For the in-house assays, we assumed that the PCR assays were not performed on the same thermocycler as described by the authors. We clearly observed that specificity decreased when sensitivity increased. In order to qualify a stool sample prior to FMT, both high sensitivity and specificity are required. The combination of the qPCR methods of Poirier et al. and Stensvold et al. for confirmation allowed the sensitivity to be increased up to 87% (Supplementary Figure S1), but the specificity decreased at the same time to 94%. Sequencing of amplified products would therefore be necessary to confirm all positive samples.

Interestingly, the detection of ST4 was lower with DLM, as previously described [13], but also with some qPCR assays. Except for ST3, for which no significant difference was observed, the number of specimens was too low to conclude for the other STs. The targeted regions within the 18S rRNA gene are different for the three in-house qPCR assays. Diversity between the 18S rRNA gene sequence of *Blastocystis* STs is known to be important, which is the reason why it is challenging to design primers and probes that are able to detect all of them. For *Blastocystis* subtyping, most studies use a 600-bp barcode region of the 18S rRNA gene described by Scicluna et al. [21]. However, the Poirier et al. qPCR technique also allows ST identification, as it targets a sufficiently large region that includes variable sequences between STs, which was not the case for the other techniques [13]. So, when this PCR was negative, the qPCR product was sequenced to confirm it was a true positive, although it was not possible to specify the ST, which is a limitation of our study. In addition, we cannot exclude the presence of co-infection by different STs among positive stools included in our study, and it would be more accurate to realize a cloning before sequencing of the 18S rDNA barcode region in order to highlight these possible co-infections.

In our hands, the Dagci et al. qPCR presented the lowest sensitivity of the qPCR assays, and the three other qPCR assays had comparable performances even though none achieved complete satisfaction or stood out as the best tool to recommend (Table 1). For clinical biologists, the choice will depend on (i) equipment availability (Allplex^TM^ Gastrointestinal Panel-Parasite Assay needs a CFX96^TM^ platform, contrary to the in-house assays, which could be adapted on various real-time PCR platforms, see Table 2), (ii) technical skills (commercialized assays are often easier to prepare), and (iii) clinician expertise (positive/negative result is directly provided on the final report for the commercialized assay without curve amplification interpretation need).

For medical diagnosis, detection of PCR inhibitors is an important issue, especially for complex specimens such as stools. This can be achieved by the use of internal controls for probe-based qPCR, or by spiking positive control in the DNA extract of a patient for SYBR green-based qPCR. However, the latter is time consuming, and less practical for medical practice. Moreover, the standardization of commercial kits fits better with the quality policy required in medical laboratories, and the CE-IVD label is interesting.

Considering all these characteristics, the Poirier et al. qPCR method seems more adapted to research use, as it would be cheaper and allows *Blastocystis* subtyping by sequencing PCR products. The Allplex^TM^ Gastrointestinal Panel-Parasite Assay and the Stensvold et al. qPCR could be preferred in diagnostic laboratories, depending on the habits and the convenience of the technical and medical teams. As mentioned above, the clinical context of the patient(s) (such as FMT) should also be considered when using the Allplex^TM^ Gastrointestinal Panel-Parasite Assay, as its specificity is lower than the Stensvold et al. qPCR and may require a second technique for confirmation.

## Figures and Tables

**Table 1:** Sensitivity and specificity of the four qPCR assays and direct light microscopy.

	Poirier et al. [12]	Stensvold et al. [14]	Dagci et al. [15]	Allplex^TM^ GIPPA ^1^	Direct Light Microscopy
Sensitivity	0.71	0.79	0.55	0.84	0.34
Specificity	0.98	0.94	1.00	0.82	1.00

^1^ Gastrointestinal Panel-Parasite Assay.

**Table 2:** Summary of important characteristics of the four qPCR assays tested.

	Poirier et al. [12]	Stensvold et al. [14]	Dagci et al. [15]	Allplex^TM^ GIPPA ^1^
Probe-based qPCR	n.a. ^2^	Taq-Man probe	Taq-Man probe	TOCE^TM^ technology ^3^
Internal control	n.a. ^2^	yes	not designed in the article	yes
CE-IVD marking ^4^	n.a. ^2^	n.a. ^2^	n.a. ^2^	yes
Subtyping on amplicons	yes	n.a. ^2^	n.a. ^2^	n.a. ^2^

^1^ Gastrointestinal Panel-Parasite Assay; ^2^ not applicable; ^3^ Tagging Oligonucleotide Cleavage and Extension; ^4^ indicates the in vitro diagnostic (IVD) device complies with the European in vitro diagnosic devices directive.

## Supplementary Materials

The following are available online at https://www.mdpi.com/2076-2607/8/11/1768/s1: Figure S1: Sensitivity and specificity of different combined methods. Table S1: Results of direct light microscopy and qPCR assays according to extraction methods and sequencing. GIPPA: Gastrointestinal Panel-Parasite Assay.
