# Supplementary figures and images for: Comparison of DNA Extraction Methods and Real-Time PCR Assays for the Detection of Blastocystis sp. in Stool Specimens
Source: Microorganisms. 2020 Nov 11;8(11):1768. doi: 10.3390/microorganisms8111768 (PMC7696706; doi:10.3390/microorganisms8111768)

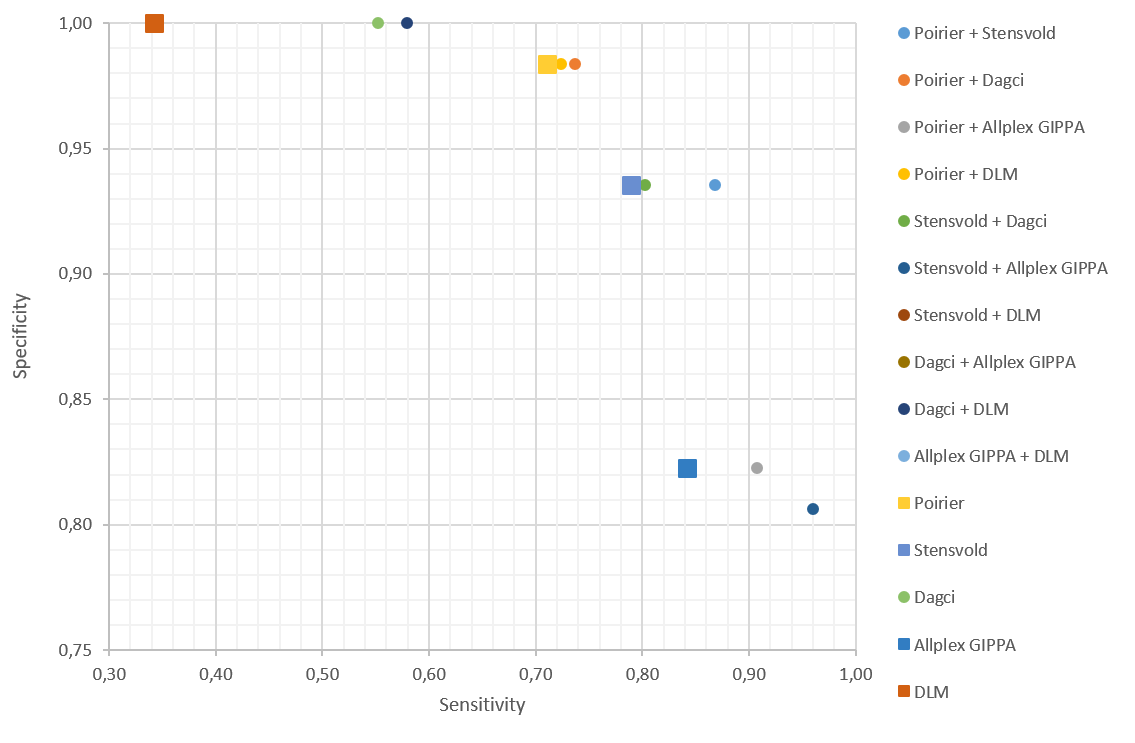

Supplement: Supplementary file 1 [file microorganisms-08-01768-s001.zip › Supplementary files/Supp Fig S1.png]
